# A Web-Based Well-being Program for Health Care Workers (Thrive): Protocol for a Randomized Controlled Trial

**DOI:** 10.2196/34005

**Published:** 2022-04-21

**Authors:** Luke A Egan, Mary Mulcahy, Karen Tuqiri, Justine M Gatt

**Affiliations:** 1 Neuroscience Research Australia Randwick Australia; 2 Person Centred Care The Prince of Wales Hospital Randwick Australia; 3 The Prince of Wales Hospital Randwick Australia; 4 School of Psychology University of New South Wales Sydney Australia

**Keywords:** well-being, Composure, Own-worth, Mastery, Positivity, Achievement, and Satisfaction for Wellbeing, COMPAS-W, mental health, resilience, health care, hospital, brain, neuroscience, online, randomized controlled trial, RCT

## Abstract

**Background:**

Mental health has come to be understood as not merely the absence of mental illness but also the presence of mental well-being, and recent interventions have sought to increase well-being in various populations. A population that deserves particular attention is that of health care workers, whose occupations entail high levels of stress, especially given the ongoing COVID-19 pandemic. A neuroscience-based web-based well-being program for health care workers—the Thrive program—has been newly developed to promote habits and activities that contribute to brain health and overall mental well-being.

**Objective:**

This paper describes the protocol for a randomized controlled trial whose objective is to evaluate the Thrive program in comparison with an active control condition to measure whether the program is effective at increasing well-being and decreasing symptoms of psychological distress in health care workers at a designated Australian hospital.

**Methods:**

The trial will comprise two groups (intervention vs active control) and 4 measurement occasions over a 12-week period. A survey will be administered in each of weeks 0, 4, 8, and 12, and the well-being program will be delivered in weeks 1-7 (via web-based video presentations or digital pamphlets). Each of the 4 surveys will comprise a range of questionnaires to measure well-being, psychological distress, and other key variables. The planned analyses will estimate group-by-time interaction effects to test the hypothesis that mental health will increase over time in the intervention condition relative to the active control condition.

**Results:**

The Thrive program was delivered to a small number of wards at the hospital between February 2021 and July 2021, and it will be delivered to the remaining wards from October 2021 to December 2021. A power calculation has recommended a sample size of at least 200 participants in total. A linear mixed model will be used to estimate the interaction effects.

**Conclusions:**

This trial seeks to evaluate a new web-based well-being program for health care workers at a major public hospital. It will contribute to the growing body of research on mental well-being and ways to promote it.

**Trial Registration:**

Australian New Zealand Clinical Trials Registry ACTRN12621000027819; https://tinyurl.com/58wwjut9

**International Registered Report Identifier (IRRID):**

DERR1-10.2196/34005

## Introduction

### Background

In recent decades, the medical and behavioral sciences have recognized that mental health is not merely the absence of mental illness but also the presence of mental well-being [1]. Thus, mental illness and well-being are distinct albeit related constructs that need to be considered in research and clinical practice. In a seminal study of the US adult population, Keyes [2,3] delineated three categories of mental well-being: flourishing, moderately healthy, and languishing. Those with high well-being and no mental illness are described as flourishing, whereas those with low well-being may be free of illness (*pure languishing*) or not (eg, *depressed and languishing* in the case of those with major depression). In the representative sample of >3000 adults, less than one-fifth were flourishing, and a comparable proportion were languishing. Of those with low well-being, over half did not meet criteria for a major depressive episode, generalized anxiety disorder, panic disorder, or alcohol dependence over the preceding 12 months. On average, those who were purely languishing reported less than one symptom of each of these disorders, suggesting that low well-being is not a proxy for subclinical illness but rather a distinct condition. This was supported by confirmatory factor analyses that indicated that a correlated 2-factor model was the best way to account for the data on mental illness and well-being. Furthermore, it was found that pure languishing was not only as prevalent as pure illness (ie, illness without low well-being) but also associated with more severe psychosocial outcomes (relative to pure illness) on 9 out of 11 measures. Clearly, low mental well-being can place a substantial burden on the individual and on society that stands apart from the costs of illness alone and needs to be targeted in its own right in health promotion strategies.

More recently, there have been important advances in the measurement and understanding of mental well-being. Gatt et al [4] developed a composite measure of well-being—the Composure, Own-worth, Mastery, Positivity, Achievement, and Satisfaction for Wellbeing (COMPAS-W) scale—that assesses both hedonia (ie, positive affect and life satisfaction) and eudaimonia (ie, fulfillment of one’s potential and having a sense of life purpose) across six subcomponents: Composure (dealing effectively with stress or adversity), Own-worth (a sense of autonomy and self-respect), Mastery (self-confidence and perceived control over one’s environment), Positivity (happiness and optimism), Achievement (setting and pursuing goals), and Satisfaction (with one’s life, health, and relationships). This factor structure was confirmed in a major twin study of Australian adults, as was the scale’s reliability and validity. For example, well-being was associated with lower levels of depression, anxiety, and stress symptoms; higher work productivity; and healthier diet, exercise, and sleep habits [4]. The COMPAS-W measure has allowed researchers to explore the genetic and neural underpinnings of mental well-being [5-7] and, crucially, to determine the extent to which well-being might be modifiable through interventions and other environmental influences. From their twin study, Gatt et al [4] derived heritability estimates between 24% and 48% across the 6 subcomponents of well-being, with the remaining variance attributed to the unique environment. This indicates that, although well-being is significantly influenced by genetic factors, it is certainly subject to external influences as well, which is encouraging for practitioners who would seek to raise levels of well-being in a given population.

As mental well-being has been recognized as a key construct in its own right and one that is substantially determined by nongenetic factors, it has become the focus of interventions that aim to elevate it or at least prevent its decline. To date, there have been 2 randomized controlled trials (RCTs) investigating the effects of an intervention on COMPAS-W scores. In a trial involving a subsample from the aforementioned twin study, Routledge et al [8] evaluated a 30-day *brain training* (ie, computerized cognitive training) program comprising games and exercises related to cognitive and affective processing as well as emotional regulation. Relative to a waitlist control group, training participants exhibited changes in explicit emotion identification and implicit emotion bias for a number of facial expressions, but these alterations did not lead to changes in well-being or psychological distress. However, the brain training program did not exclusively target mental health but rather comprised a broader set of cognitive and emotional activities, and many participants did not complete the minimum amount of training recommended by the researchers. Furthermore, the recruitment process excluded participants with current or lifetime psychiatric disorders or substance abuse. Therefore, there may have been a ceiling effect whereby many participants were already functioning well enough that they had little to gain from the intervention in that regard, especially if they did not complete the recommended amount of training.

In the second RCT study, Chilver and Gatt [9] evaluated a 6-week positive psychology intervention relative to an active control condition in 326 university students. They found that the intervention led to significant increases in subjective well-being and—in those who had low resiliency resources at the outset—increases in composite well-being and decreases in psychological distress (ie, depression and anxiety symptoms). Again, this is consistent with the possibility that a ceiling effect exists to some degree whereby those who are more mentally healthy at the outset have less to gain from an intervention. However, across all the participants, the intervention by Chilver and Gatt [9] resulted in significant increases in the well-being subcomponent of Satisfaction, suggesting that certain aspects of well-being may be more responsive to training even in those with higher resilience at baseline. Together, these studies suggest that it is possible to improve well-being outcomes via particular intervention approaches and that they may be more efficacious in populations that are less resilient or more exposed to stress and other kinds of adversity.

One such population to target is hospital-based health care workers, who work under demanding and stressful conditions. There is extensive literature on nurses’ experiences in the workplace, highlighting a range of demands and hardships that can compromise staff well-being. For instance, in a survey of >40,000 nurses from >700 hospitals across 5 countries, Aiken et al [10] found that the proportion of nurses who reported job dissatisfaction was >40% in the United States; >30% in Canada, England, and Scotland; and approximately 20% in Germany. For comparison, the researchers pointed out that job dissatisfaction in the general population of American professionals was only 10%, indicating that dissatisfaction was markedly higher among nurses. Furthermore, a large proportion (15%-40% depending on the country) of the surveyed nurses reported that they were planning to leave their job in the following year. In a more recent survey of >3000 Australian health care workers, >70% stated that their workloads exceeded what they were capable of doing well at least once or twice a week, and >25% stated that they were thinking of leaving their profession [11]. The typical sources of psychological distress reported by health care workers include heavy workloads, time pressures, role ambiguity, lack of control or flexibility, and lack of participation in decision-making [12].

The COVID-19 pandemic has placed additional demands on health care workers [13]. In Australia, a survey of 637 primary health care nurses [14] revealed that most respondents (nearly 75%) did not always have access to sufficient personal protective equipment, almost half had concerns about a lack of support from their employers, and over one-third stated that the quality of care at their workplace had diminished (at least slightly) since the start of the pandemic. In addition, the government of New South Wales (Australia’s most populous state) published a summary of worldwide research on the impact of the pandemic on the mental health of health care workers [15], finding that the pandemic had placed these workers at increased risk of psychological distress, including anxiety about contracting the virus. Of 433 Australian health care workers surveyed, approximately 50% reported increased workloads, anxiety, and tiredness, and >70% reported higher stress at work.

The mental well-being of nurses and other health care workers is vitally important not only for the workers themselves but also for the health and safety of their patients. Structural equation models have suggested that well-being mediates the relationship between organizational factors and patient care. For example, a study of 324 Hong Kong nurses [16] found that nurses’ perceptions of support from their workplace, supervisor, and colleagues predicted their psychological well-being, which in turn predicted their safety performance. Similarly, a study of 345 Iranian nurses [17] found that organizational support predicted psychological well-being, which in turn predicted both job satisfaction and quality of care, and a study of 474 Taiwanese nurses [18] concluded that well-being predicted safety attitudes. In addition, a study of 325 Pakistani nurses found that those with higher levels of negative emotion were judged by their peers to engage in higher levels of “deviant” workplace behavior such as deliberately arriving late to work or taking undeserved breaks to avoid doing work, which could compromise patient care in numerous ways [19]. Furthermore, in a study of 637 US nurses, Dyrbye et al [20] observed that those who scored lower on a well-being index were more likely to have made a patient care error in the previous 3 months. Although the relationship between well-being and patient care may go in either direction (or be attributable to a third variable), it is eminently plausible that low well-being (and the psychosocial dysfunction that comes with it) can result in suboptimal job performance and more frequent errors (cf Keyes [3], who found that lower well-being was related to higher absenteeism and reduced workdays, and Gatt et al [4], who found that lower well-being was associated with higher absenteeism and lower productivity on the job). Clearly, there are substantial benefits that may be gained by addressing shortfalls in health care workers’ well-being.

Fortunately, there is some research to suggest that interventions for health care workers can lead to reductions in stress and improvements in mental well-being [21], although the evidence base is relatively limited at present. For instance, Orly et al [22] administered a cognitive behavioral intervention for nurses that resulted in benefits related to stress and mood, but the sample size was small (N=36), the participants were not randomly allocated to the intervention and control conditions, and there was no follow-up (ie, no testing beyond the end of the intervention). Tveito and Eriksen [23] conducted an RCT of a health and fitness program for nursing home staff, finding benefits in terms of self-reported health and stress but, again, the sample was small (N=29), and there was no follow-up. Similarly, Daigle et al [24] conducted an RCT of a mindfulness-based stress-reduction program for nurses, finding improved mood in the intervention group but, yet again, the sample was small (N=52), and there was no follow-up. Similarly, Bolier et al [25] carried out an RCT of a web-based mental health program for health care workers, but the randomization was conducted at the ward level rather than the individual level and, of the 178 participants allocated to the intervention group, only 9 actually engaged in the web-based activities. Hence, our study seeks to address some of the gaps in the literature on well-being programs for health care workers.

This paper outlines the protocol for an RCT evaluating the effects of a new well-being program—the *Thrive* program—for health care workers at a large public hospital in metropolitan Sydney, New South Wales, Australia. Thrive is a web-based psychoeducational program that provides information and guidance within seven areas of life related to mental well-being: sleep, exercise, nutrition, stress management, social connection, cognitive challenge, and life purpose. The program is innovative in that it covers how these 7 aspects of life affect not only well-being in general but also brain health in particular. With advances in neuroscience and neuroimaging in the 21st century, there is now a sufficient knowledge base to underpin this specialized intervention, which is aimed at promoting well-being via healthy habits and lifestyle decisions that directly affect the organ on which mental health ultimately depends—the brain. Another key strength of this study is that the COMPAS-W well-being scale [4] will be used as the primary outcome measure, allowing for a comprehensive assessment of the participants’ subjective and psychological well-being and an opportunity to detect whether one or more of the 6 subcomponents of well-being is more or less responsive to the intervention. To the authors’ knowledge, this will be the first intervention for health care workers to use an empirically derived measure of well-being that has also been validated with regard to genetic [7,26-28] and neural [5,6,29] markers as well as psychological factors, including resilience, trauma, and coping strategies [30,31].

### Objectives

This study has 2 main objectives. First, it aims to determine whether the Thrive program results in significant gains in well-being—as measured by the COMPAS-W scale—in the intervention group compared with an active control group and, if so, which of the specific COMPAS-W subcomponents is most responsive to the program. Second, from the baseline measurements, the study will inform researchers, hospital staff, and other stakeholders of the current levels of overall mental health and well-being among the health care workers at the target hospital.

## Methods

### Design

**Figure 1:**
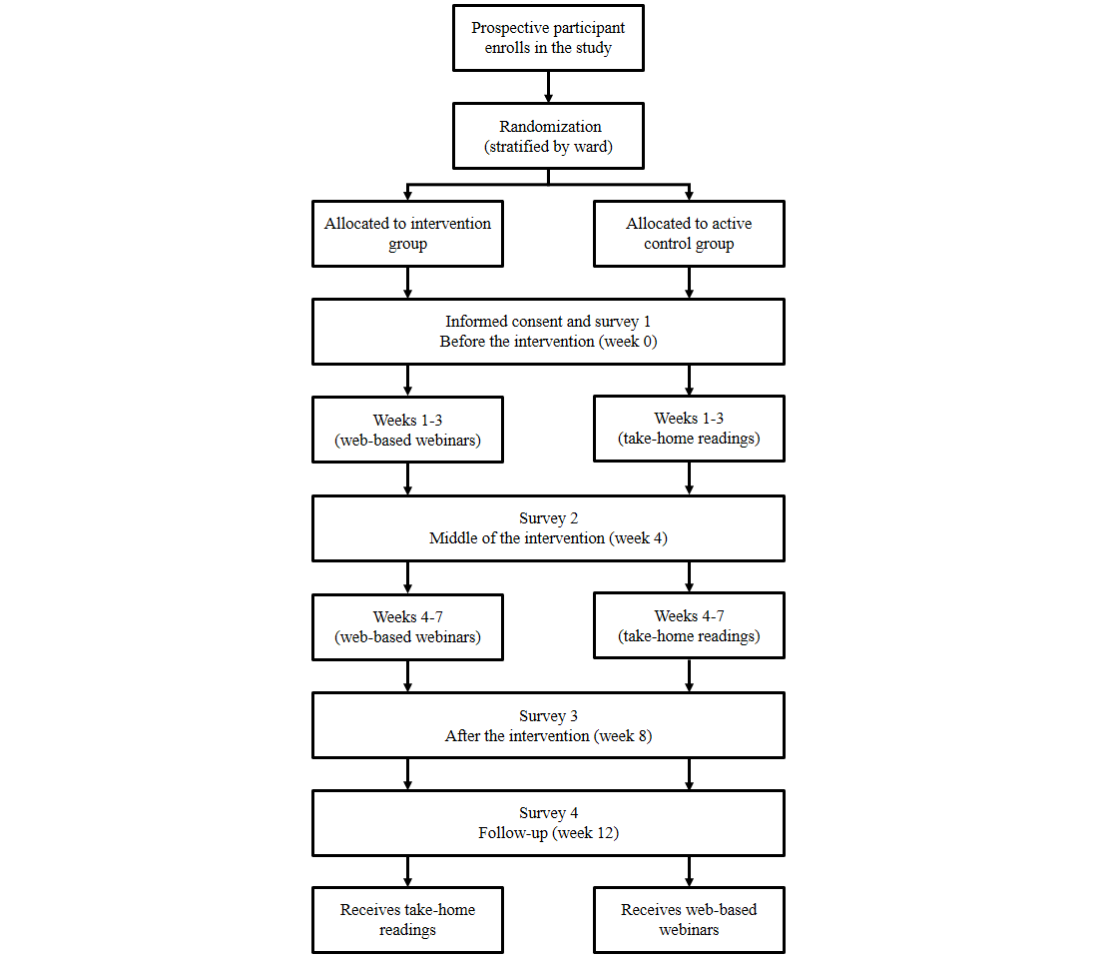
Study timeline.

The Thrive program will be evaluated in an RCT with 2 conditions and 4 measurement occasions. Thus, there will be two main independent variables—one between-group factor and one repeated-measure factor—as well as a selection of covariates and dependent variables. The two conditions will be the intervention (ie, the Thrive webinar program) and an active control condition (only an abbreviated pamphlet version of the educational components of the program). The study will take place over a 12-week period, and a web-based survey will be administered at each of the 4 measurement occasions. In week 0 (ie, 1 week before the program commences), the participants will complete the baseline survey (before the intervention). The Thrive program (and the parallel active control program) will run for 7 weeks, from week 1 to week 7. The participants will complete the second survey in week 4 (at the midpoint of the program), the third survey (after the intervention) in week 8 (ie, 1 week after the conclusion of the program), and the fourth survey (follow-up) in week 12 (ie, 4 weeks after the intervention). Each survey will take approximately 20 to 30 minutes to complete. Once all 4 surveys have been administered and the study period has concluded, all the participants in the active control condition will be given access to the full version of the Thrive program. The study timeline is illustrated in Figure 1.

### Participants

The participants will be health care workers from the Prince of Wales Hospital (POWH), a large public hospital in metropolitan Sydney, New South Wales, Australia. The participants will be recruited via flyers displayed around the hospital, emails sent to hospital staff mailing lists, and announcements from management (eg, Nursing Unit Managers). All health care workers from the hospital (eg, nurses, physicians, and allied health professionals) will be welcome to sign up for the study.

### Eligibility Criteria

Prospective participants will be eligible to enroll in the study provided that they are health care workers at POWH, comfortable using written English to complete the program and surveys, willing to take four 20-to-30–minute web-based surveys across the 12-week study period, willing to complete the 7-week well-being program, and able to access the internet for the surveys and program content.

### Recruitment

Study advertisements will invite POWH staff to sign up for the 12-week study and notify them that participants will be randomly assigned to one of two conditions, labeled *online presentations* and *take-home readings*. Those assigned to the former condition will be the intervention group, and those assigned to the latter condition will be the active control group. Once assigned, the participants will know whether they are in the *online presentations* or *take-home readings* condition, but they will be blind to which condition is the true intervention versus the active control. They will also be assured that all participants in one version of the Thrive program will receive all the resources of the other version after the end of the 12-week study period. This assurance should reduce the incentive for participants in one group to share any course content with a participant from the other group (the participants will also be explicitly instructed not to share anything with anyone else throughout the program).

Potential participants will be informed that those who complete the Thrive program will be eligible to receive Continuing Professional Development (CPD) points. At POWH, nurses and other health care workers are expected to engage in ongoing professional development by attending workshops, completing courses, and participating in other such activities, with a certain number of CPD points awarded for each activity. Each worker has to earn at least 20 points per year, and each Thrive participant will receive 10 points after completing the program. As the control participants will gain access to the full Thrive program at the end of the 12-week study period, all the participants will receive 10 points regardless of the condition to which they are assigned. There are numerous ways outside the Thrive program in which a given health care worker can earn their required CPD points, so the offer of 10 points for completing the program is not an undue inducement to participate but rather fair compensation for the participants’ time and effort. Each participant will also receive a Certificate of Completion from the research team.

### Randomization and Informed Consent

Hospital staff interested in participating will sign up for the study by writing their details (ie, name, email address, and ward) on registration sheets posted around the wards, by registering on the web via the hospital’s professional development website, or by emailing a designated member of the research team (MM). The final list of participants will be sorted according to ward to allow for stratified randomization. For each ward, a random number generator will be used to allocate half the participating staff to the intervention condition and half to the active control condition. Next, the participants will be notified by email as to the condition to which they have been assigned. This email will also provide the Participant Information Statement and Consent Form and a link to the first survey. After reading the form, those who are willing to participate will click on the link to access the first survey. The first item of this survey provides a button whereby the participant can digitally record their consent to participate. For the remainder of the study period, the participants will be sent the relevant survey links and program resources by email at the appropriate times.

All participants will be free to withdraw at any time from the surveys, the Thrive program, or both the surveys and the program without penalty and without having to give a reason, with the provision that only those who complete the Thrive program will receive the 10 CPD points and Certificate of Completion. However, completion of the surveys will not be required to earn the points and certificate. In other words, if a participant is enjoying the program and wishes to finish it but does not want to continue providing data via the surveys, they will be welcome to do so.

### Sample Size

Power calculations suggest that at least 200 participants need to be recruited (100 per condition) to detect a small group-by-time interaction effect at 80% power. This sample size was calculated using the statistical software package G*Power (version 3.1.9.2) [32,33]. Assuming a small effect of 0.01 (partial *η*^2^), a conventional type-1 error rate of 0.05, and a correlation of 0.5 among the repeated outcome measures, the study will be able to detect such an effect at 80% power with 69 participants in each group. However, considering an attrition rate of up to 30% by the final measurement occasion, it will be necessary to recruit at least 99 people per group, so we will aim for a total sample size of at least 200. The projected attrition rate of 30% is based on the attrition rates reported in previous RCTs [23-25] that examined interventions to increase well-being in hospital staff. Additional power calculations show that the study will be able to detect effects as small as 0.04 (partial *η*^2^) even under the strictest nonsphericity correction and possible attrition of up to 65%.

### Procedure and Materials

#### Intervention

The intervention participants will receive the full version of the Thrive program, comprising a 1-hour web-based presentation each week for 7 weeks. Each presentation will be a webinar (web-based seminar) delivered via the Zoom platform (Zoom Video Communications Inc). Participants will view the webinars as prerecorded videos on a dedicated webpage, which will ensure uniform delivery of the intervention. Each webinar will be copresented by 2 members of the research team. One presenter (MM) is a Nurse Educator from the hospital with a Master’s degree in Adult Education and extensive experience in providing professional development courses to health care workers. The other presenter (LAE) is a researcher from the Gatt Group at Neuroscience Research Australia (NeuRA) and the University of New South Wales (UNSW) with a PhD in Psychology and extensive experience in lecturing, tutoring, and counseling.

Each webinar will follow a 3-step sequence named *Inform, Inspire, Improve*. The *Inform* and *Inspire* sections will last approximately 25 minutes each, and the *Improve* section will last approximately 10 minutes. During the *Inform* section, the presenters will share scientific findings from the fields of neuroscience, medicine, and psychology to educate the participants on the links among brain health, mental well-being, and one of the 7 areas of life covered by the program. This section will provide knowledge only, as a background to the subsequent sections. During the *Inspire* section, the presenters will provide advice, guidance, and recommendations on how to improve one’s brain health and mental well-being in the relevant area of life. This section will build upon the preceding knowledge by offering practical suggestions to implement in one’s daily life. Finally, during the *Improve* section, the webinar viewers will have an opportunity to reflect on the knowledge and suggestions shared in the preceding sections, and they will be invited to commit to enacting at least one of the suggestions in their own lives.

The knowledge and recommendations provided in each webinar are based on peer-reviewed empirical studies from the medical and behavioral sciences, with a special emphasis on neuroscientific findings. The webinars also draw upon other authoritative sources such as the US Department of Health and Human Services, the Australian Government National Health and Medical Research Council, and the World Health Organization. The full reference list for the Thrive program contains >250 peer-reviewed publications, and the participants will be provided with a reference list for each week of the program in case they wish to check anything or explore the literature themselves. Textbox 1 provides an overview of the program content for each of the 7 webinars.

**Overview of the Thrive webinar content for the intervention group.**

**Webinar topics and content**

- Week 1 (sleep): impact of sleep quantity and quality on well-being and the brain; tips for improving sleep quality in relation to light exposure, exercise, meal timing, and nutrition; cognitive behavioral tips for sleeping better; and accessing treatment for a sleep-related disorder
- Week 2 (exercise): impact of physical activity on well-being and the brain; tips for increasing motivation to exercise in relation to social connection, natural environments, and novelty or variety; using the internet to find new ways to exercise; exercising in a way that suits one’s personal preferences; and how to access an exercise physiologist through the publicly funded health care system
- Week 3 (nutrition): impact of nutrition on well-being and the brain; links among nutrition, human evolution, and chronic noncommunicable diseases; tips for improving one’s diet in relation to avoiding empty calories, practicing mindful eating, and accommodating dietary restrictions; and how to get tested for micronutrient deficiencies
- Week 4 (stress management): impact of stress on well-being and the brain; how the stress system works; tips for managing and reducing stress using mindfulness, controlled breathing, and effective coping strategies; and how to access treatment for posttraumatic stress disorder or a stress-related disorder
- Week 5 (social connection): impact of social connections on well-being and the brain; tips for improving connections using compassionate communication, active listening, nonverbal communication, and acts of kindness; and how to use the internet to make new social connections
- Week 6 (cognitive challenge): impact of cognitive challenge on well-being and the brain; tips on challenging oneself using new knowledge, linguistic or mathematical puzzles, skill development, and novelty; and how to overcome anti-intellectual stigma and follow one’s curiosity
- Week 7 (life purpose and meaning): impact of life purpose on well-being and the brain; the differences between hedonia and eudaimonia; the state of flow and how to achieve it; and tips for achieving a greater sense of purpose and fulfillment by targeting character strengths and weaknesses, setting meaningful goals, accepting unavoidable hardship, and identifying one’s core values

Each week, in addition to the reference list, the intervention participants will receive a 1-page *reflective sheet* and a 4-page infographic summary. The reflective sheet will prompt participants to write down their key learning from that week’s webinar, what this learning means to them, and what they will do next to act on it. After viewing each webinar, the participants will be encouraged to fill in their reflective sheet and add it to their CPD portfolio, although this is not a requirement to complete the program. The infographic document will provide a summary of the *Inform* section of that week’s webinar (ie, a summary of the neuroscience and other background information covered in the webinar).

#### Active Control

The active control participants will receive an extremely reduced version of the Thrive program. In each of the 7 weeks, they will be emailed the infographic summary on that week’s topic and instructed to read it. The infographic documents will be identical to those received by the intervention participants. In short, the control participants will be provided with only the *Inform* section for each week’s topic, in the form of the infographic summary only. They will not receive any tips and advice for promoting well-being. Thus, the intervention participants will receive knowledge plus practical advice and reflective opportunities (in webinar and document forms), whereas the control participants will receive knowledge only (in document form only). The control participants will receive a reference list to accompany each infographic summary, but they will not receive the reflective sheets. To summarize, each week, the intervention participants will receive a webinar, reflective sheet, infographic, and reference list, whereas the active control participants will receive the infographic and reference list only.

### Survey Measures

#### Overview

A range of questionnaires will be administered via the web-based surveys, but not all will be administered at each measurement occasion (see Table 1 for an outline of the questionnaire timings).

**Table 1:** Questionnaires delivered at each time point.

Questionnaire	Survey 1 (week 0)	Survey 2 (week 4)	Survey 3 (week 8)	Survey 4 (week 12)
Demographics	✓			
Medical history	✓			
Health and lifestyle	✓	✓	✓	✓
COMPAS-W^a^	✓	✓	✓	✓
RRC-ARM^b^	✓			
DASS-21^c^	✓	✓	✓	✓
Abbreviated POMS^d^	✓	✓	✓	✓
Brief COPE^e^	✓	✓	✓	✓
Self-Compassion Scale	✓			✓
Compassion Scale	✓			✓
MAAS^f^	✓			✓
HPQ^g^ (work performance items)	✓			✓
UWES^h^	✓			✓
DLE^i^ (trauma items)	✓			
DLE (COVID-19 items)	✓	✓	✓	✓
COVID-19 Exposure Survey	✓	✓	✓	✓
Thrive attendance items		✓	✓	✓
Thrive satisfaction items			✓	✓

^a^COMPAS-W: Composure, Own-worth, Mastery, Positivity, Achievement, and Satisfaction for Wellbeing Scale.

^b^RRC-ARM: Resilience Research Centre Adult Resilience Measure.

^c^DASS-21: Depression, Anxiety, and Stress Scale–21-item version.

^d^POMS: Profile of Mood States.

^e^COPE: Coping Orientation to Problems Experienced.

^f^MAAS: Mindful Attention Awareness Scale.

^g^HPQ: Health and Work Performance Questionnaire.

^h^UWES: Utrecht Work Engagement Scale.

^i^DLE: Daily Life Events.

#### Demographics

This questionnaire will measure age, sex, education, occupation, and other demographic characteristics.

#### Medical History

This questionnaire will ask whether the participant or anyone in their immediate family has ever been diagnosed with a learning or developmental disorder or diagnosed with and treated for a psychological or psychiatric disorder.

#### Health and Lifestyle

This questionnaire will ask about health-related habits, including questions on diet, exercise, sleep, and drug and alcohol use.

#### COMPAS-W Scale

The COMPAS-W scale [4] is a 26-item questionnaire that measures overall mental well-being as well as the six subcomponents of Composure, Own-worth, Mastery, Positivity, Achievement, and Satisfaction. Each item presents a statement with a 5-point response scale ranging from *strongly disagree* to *strongly agree*. The respondents are asked to answer the items in terms of how they feel *most of the time* but, in this study, the instructions will be modified for the second, third, and fourth surveys to ask the respondents how they have felt *over the past month* (ie, since taking the previous survey).

#### Resilience Research Centre Adult Resilience Measure

The Resilience Research Centre Adult Resilience Measure [34] is a 28-item questionnaire that measures resiliency resources (eg, *I know where to get help in my community*). Each item presents a statement with a 5-point response scale ranging from *not at all* to *a lot*. This scale does not specify a period but simply asks the respondents to what extent each statement describes them.

#### Depression, Anxiety, and Stress Scale–21-Item Version

The Depression, Anxiety, and Stress Scale–21-item version [35,36] is a 21-item questionnaire that measures psychological distress (ie, symptoms of depression, anxiety, and stress). Each item presents a statement with a 4-point response scale ranging from *did not apply to me at all* to *applied to me very much or most of the time*. The respondents are asked to answer each statement in terms of how much it has applied to them *over the past week*.

#### Abbreviated Profile of Mood States (Revised Version)

The revised version of the Abbreviated Profile of Mood States [37] is a 40-item list of adjectives describing positive and negative mood states (eg, vigorous or unhappy), with a 5-point response scale ranging from *not at all* to *extremely*. The respondents are asked to what extent each adjective describes how they feel *right now*.

#### Brief Coping Orientation to Problems Experienced

The Brief Coping Orientation to Problems Experienced scale [38] is a 28-item questionnaire that measures 14 specific coping styles across the broader categories of approach coping (eg, *I’ve been taking action to try to make the situation better*) and avoidant coping (eg, *I’ve been refusing to believe that it has happened*). The 14 subscales are named *active coping*, *planning*, *positive reframing*, *acceptance*, *humor*, *religion*, *using emotional support*, *using instrumental support*, *self-distraction*, *denial*, *venting*, *substance use*, *behavioral disengagement*, and *self-blame*. Each item presents a method of coping with a 4-point response scale ranging from *I haven’t been doing this at all* to *I’ve been doing this a lot*. The respondents are asked to answer each statement in terms of how much they have been using that coping style *in the past month*.

#### Self-Compassion Scale

The Self-Compassion Scale [39] is a 26-item questionnaire that measures self-compassion across six subscales named *self-kindness*, *self-judgment*, *common humanity*, *isolation*, *mindfulness*, and *overidentification*. Each item presents a statement with a 5-point response scale ranging from *almost never* to *almost always*. The respondents are asked to answer each statement in terms of how they *typically* act toward themselves in difficult times.

#### Compassion Scale

The Compassion Scale [40] is a 16-item questionnaire that measures compassion for others across four subscales named *kindness*, *common humanity*, *mindfulness*, and *indifference*. Each item presents a statement with a 5-point response scale ranging from *almost never* to *almost always*. The respondents are asked to answer each statement simply in terms of *how often* they feel or behave in the stated manner.

#### Mindful Attention Awareness Scale

The Mindful Attention Awareness Scale [41,42] is a 15-item questionnaire that measures mindfulness (ie, nonjudgmental consciousness of the present moment). Each item presents a statement with a 6-point response scale ranging from *almost never* to *almost always*. The respondents are asked to what degree each statement pertains to their *current experience*.

#### Health and Work Performance Questionnaire Scales (Employee Version)

Two sections from the employee version of the World Health Organization Health and Work Performance Questionnaire [43]—scale B9 and scale B12—will be used to measure work performance. Scale B9 contains 5 items asking how many days in the past 4 weeks the respondent missed an entire workday, missed part of a workday, or performed extra work outside their usual working hours. Scale B12 contains 7 items about the respondent’s work performance in the past 4 weeks (referring to both overperformance and underperformance), with a 5-point response scale ranging from *none of the time* to *all of the time*.

#### Utrecht Work Engagement Scale

The Utrecht Work Engagement Scale [44] is a 17-item questionnaire that measures the respondent’s level of engagement with their work (eg, *I am enthusiastic about my job*). Each item presents a statement with a 7-point response scale ranging from *never* to *always/every day*.

#### Daily Life Events

The Daily Life Events (DLE) scale [45] is a list of minor and major positive and negative life events, and the respondents are asked whether each event has happened to them and, if so, whether it has had a positive, neutral, or negative impact on them. For this study, we used the trauma items from the original DLE scale and adapted the other items to be answered in reference to COVID-19. The trauma section lists 7 potentially traumatic life events (eg, being physically or sexually assaulted) and asks the respondents whether each event has ever happened to them and, if so, how many years ago it last occurred. The COVID-19 section lists 15 life events (eg, separation from family and working from home) and asks the respondents whether each event has happened to them in the last 12 months (ie, during the COVID-19 pandemic) and, if so, how many months ago it last occurred and whether it had a positive, neutral, or negative impact on them, with a 7-point response scale ranging from −3 (*extremely negative impact*) to +3 (*extremely positive impact*). The DLE scale is provided in Multimedia Appendix 1.

#### COVID-19 Exposure Survey

The COVID-19 Exposure Survey is a compilation of items regarding the impact of the COVID-19 pandemic on the respondents and their workplace (ie, their ward at the hospital). The survey includes questions on how many times the respondent has been tested for COVID-19, whether they have contracted the virus, whether they have had to care for a patient with the virus, whether the pandemic has affected their workload, and whether they have considered leaving their job during the pandemic. The COVID-19 Exposure Survey is provided in Multimedia Appendix 1.

#### Thrive Program Attendance and Satisfaction Survey

This survey asks the respondents which of the 2 versions of the Thrive program they received (this question will serve as a manipulation check), how much of the program they have completed (ie, their attendance), whether they enjoyed it, and how helpful they felt it was for both themselves and their patients. This survey will be administered only after the Thrive program has concluded (ie, in weeks 8 and 12); however, items 7 and 8 of the survey (Multimedia Appendix 1) will be administered in week 4 to measure attendance up to that point. When item 8 is administered in week 4, it will ask about attendance only for weeks 1 to 3. When administered in weeks 8 and 12, this item will ask about attendance across all 7 weeks of the program.

### Primary and Secondary Outcomes

The primary outcomes for this RCT will be the participants’ levels of well-being (as measured by the COMPAS-W) and their levels of psychological distress (as measured by the Depression, Anxiety, and Stress Scale–21-item version). Thus, the primary outcomes will encompass both dimensions of mental health: mental well-being and mental illness. The secondary outcomes will be health and lifestyle habits, mood states, compassion (for oneself and others), mindfulness, work performance, and work engagement. Program attendance, resiliency resources, medical history, trauma, coping styles, and pandemic-related variables will serve as potential moderator variables along with demographic variables such as age, sex, ward, and occupation. The primary end point for the analysis will be the third measurement occasion (week 8), and the secondary end points will be the second and fourth occasions (week 4 and week 12).

### Anonymity and Confidentiality

Although each participant will provide their name, email address, and ward to enroll in the Thrive program, their survey responses will be completely anonymous and linked via a participant code number. At each measurement occasion, the participants will be emailed a link to the survey and, when they click on the link, they will be taken to the web-based Qualtrics (Qualtrics International Inc) platform that will be hosting the surveys. The web-based surveys will not ask for any information that could be used to identify any individual participant, and Qualtrics will not record any metadata from the respondents either. To match each participant’s responses on one measurement occasion with their responses on the other occasions, each survey will ask the respondent to provide a self-generated code. The respondent will be instructed to take the last 3 digits of their mobile phone number and the last 2 digits of their birth year to create their own 5-digit code. For example, if a participant’s phone number ended in the digits *123* and they were born in 1970, their code would be *12370*. One might argue that someone familiar with a given participant could identify their survey responses from this code, but none of the researchers who will have access to the raw data will be personally acquainted with any of the participants. Furthermore, once all the data have been collected and all the responses have been matched, the 5-digit codes will be deleted from the data set and replaced with generic, arbitrary ID numbers.

At each measurement occasion, the survey link sent to the intervention participants will be different from the link sent to the active control participants (even though the surveys themselves will be identical). This will allow the researchers to keep track of which condition each participant is in without relying on self-reports.

### Data Storage and Security

Once the data have been collected via Qualtrics, they will be downloaded directly from the Qualtrics servers onto the secure internal server at NeuRA. Only the research team will have access to the data. There will be no intermediaries (either human or technological) between Qualtrics and the research team.

### Analysis

Missing values in the data set will be estimated via multiple imputation, and the initial analyses will be conducted on an intention-to-treat basis followed by per-protocol analyses to account for levels of treatment compliance (eg, program attendance). The data will be analyzed via a linear mixed model that will allow the group-by-time interaction effects to be estimated. It is hypothesized that levels of well-being will increase over time in the intervention condition relative to the active control condition. Similarly, it is hypothesized that levels of psychological distress (ie, symptoms of depression, anxiety, and stress) will decrease over time in the intervention condition relative to the active control condition. There may also be a group-by-time interaction effect on one or more of the secondary outcomes. For example, the intervention may promote increased compassion, mindfulness, or work engagement. Furthermore, any interaction effect may itself be moderated by a variable such as resilience or coping style (cf [9,31]).

### Publication Policy

The results of this study will be shared with the hospital, the funding body, the overseeing organizations, and other relevant stakeholders. The results will also be published in peer-reviewed scholarly journals, presented at academic conferences, and publicized on the media platforms used by NeuRA and UNSW. In all publications and presentations of the findings, the data will be presented in aggregate, and no individual participants will be identifiable.

### Ethics Approval

This study has received ethical approval from the Human Research Ethics Committee of the South Eastern Sydney Local Health District (approval granted on November 9, 2020; project 2020/ETH02090), and this approval was ratified by the UNSW Human Research Ethics Committee on November 12, 2020.

## Results

The trial has been prospectively registered with the Australian New Zealand Clinical Trials Registry (registration approved on January 14, 2021; trial ID ACTRN12621000027819).

The Thrive program was developed between August 2020 and January 2021. For logistical reasons, it was decided that the program would be implemented incrementally throughout the hospital. The hospital comprises >50 wards, of which 5 were selected for the initial wave of recruitment, which occurred in December 2020 and January 2021. For the resulting cohort of participants, survey 1 was administered in February 2021, survey 2 was administered in March 2021, survey 3 was administered in April 2021, and survey 4 was administered in May 2021. The participants in the active control condition were provided with the full version of the program between May 2021 and July 2021. CPD points and certificates were awarded in August 2021. Since the successful administration of the Thrive program in the initial selection of wards, the research team and hospital management have decided to continue rolling out the program, and the staff from the remaining wards will be invited to participate in the trial from October 2021 to December 2021.

## Discussion

This study contributes to the field of research on mental well-being in a number of important ways. It addresses a population—health care workers—whose occupations entail relatively high levels of stress and burnout and who may therefore especially benefit from a staff well-being program such as Thrive. This consideration is particularly salient in light of the COVID-19 pandemic, which has confronted health care workers with additional demands and dangers. This study also addresses the methodological shortcomings of previous RCTs of interventions for health care workers with a relatively large sample, stratified random allocation, and an active control condition.

This study will furnish an evidence base for the role of a comprehensive, neuroscience-based psychoeducational program in preserving or enhancing mental well-being in health care workers. The results of the trial will be used to evaluate and refine the Thrive program so that it may be implemented effectively not only at the target hospital but also at other hospitals and potentially other settings such as corporate workplaces or educational institutions.

This trial will also contribute to the knowledge base on well-being promotion more broadly. The 21st century has seen an evolution in the understanding of mental health, revealing that a lack of illness is not enough—one also needs sufficient well-being to flourish. The Thrive program represents an attempt at putting this understanding into practice, informed by a measure of well-being—the COMPAS-W—that has been validated in terms of not only behavioral outcomes but also neural and genetic markers. It is hoped that this line of research will lead to substantial advancements in our ability to promote greater well-being—and, thus, greater mental health overall—in health care workers and beyond.

## Appendix

Multimedia Appendix 1 Daily Life Events scale, COVID-19 Exposure Survey, and Thrive Program Attendance and Satisfaction Survey.

## Abbreviations

COMPAS-W: Composure, Own-worth, Mastery, Positivity, Achievement, and Satisfaction for Wellbeing Scale
CPD: Continuing Professional Development
DLE: Daily Life Events
NeuRA: Neuroscience Research Australia
POWH: Prince of Wales Hospital
RCT: randomized controlled trial
UNSW: University of New South Wales
