# Supplementary material for: A Web-Based Well-being Program for Health Care Workers (Thrive): Protocol for a Randomized Controlled Trial
Source: JMIR Res Protoc. 2022 Apr 21;11(4):e34005. doi: 10.2196/34005 (PMC9073619; doi:10.2196/34005)
Supplement: Multimedia Appendix 1 [file resprot_v11i4e34005_app1.pdf]

Multimedia Appendix 1

A Web-Based Well-being Program for Health Care Workers (Thrive): Protocol for a Randomized Controlled Trial

doi: 10.2196/34005

Luke A. Egan[1], Mary Mulcahy[2], Karen Tuqiri[3], and Justine M. Gatt[1,4]*

[1] Neuroscience Research Australia, Randwick, New South Wales, 2031, Australia.

[2]Person Centred Care, The Prince of Wales Hospital, Randwick, New South Wales, 2031, Australia.

[3] Nursing Executive, The Prince of Wales Hospital, Randwick, New South Wales, 2031, Australia.

[4] School of Psychology, University of New South Wales, Sydney, New South Wales, 2052, Australia.

***Corresponding author:** A/Prof. Justine Gatt; Phone: +612 9399 1812; Postal address: Neuroscience Research Australia, Margarete Ainsworth Building, Barker St, Randwick, NSW, 2031, Australia; E-mail address: j.gatt@neura.edu.au

Contents

DLE (Daily Life Events) scale: Trauma Items	page 3
DLE (Daily Life Events) scale: COVID-19 Items	page 4
COVID-19 Exposure Survey	page 7
Thrive Program Attendance and Satisfaction Survey	page 10

DLE (Daily Life Events) scale: Trauma Items

Below is a list of events which may bring about changes in the lives of those who experience them.

For each event, please indicate if this kind of event has ever happened to you, and if yes, how many years ago it last occurred.

		Has it happened to you?			How many years ago did this event last occur?
1	Involvement with law enforcement<br>(e.g., being fined, arrested, detained, jailed)	Yes	No	Prefer not to answer	___________<br>years ago
2	Witnessing or experiencing a crime	Yes	No	Prefer not to answer	___________<br>years ago
3	Involvement in a war or revolution	Yes	No	Prefer not to answer	___________<br>years ago
4	Involvement in a natural disaster (e.g., bushfire, flood, other natural disaster)	Yes	No	Prefer not to answer	___________<br>years ago
5	Being personally attacked or assaulted (physical or sexual)	Yes	No	Prefer not to answer	___________<br>years ago

6	Major personal injury or illness	Yes	No	Prefer not to answer	___________ years ago
7	A stressful or upsetting event not listed above	Yes	No	Prefer not to answer	___________ years ago

DLE (Daily Life Events) scale: COVID-19 Items

Below is a list of events regardings the specific impact of COVID-19 on your current life. For this list, please consider events of the last 12 months only (i.e., during the COVID-19 pandemic only). Rate the impact of each event as judged from your current standpoint (i.e., the impact of the event in hindsight rather than at the time it occurred).

For every event, please indicate:
(1) the extent to which you view the event as having either a positive or a negative impact on your life since the event occurred, and
(2) when the event last occurred (i.e., how many months ago).

Indicate the impact of the event using the following scale:

-3 Extremely Negative Impact
-2 Moderately Negative Impact
-1 Somewhat Negative Impact
0 No Impact
+1 Slightly Positive Impact
+2 Moderately Positive Impact
+3 Extremely Positive Impact

		Impact of event								How many months ago did this event last occur?
1	Temporary separation from spouse/partner	N/A	– 3	– 2	– 1	0	+ 1	+ 2	+ 3	___________ months ago
2	Temporary separation from immediate family or household members	N/A	– 3	– 2	– 1	0	+ 1	+ 2	+ 3	___________ months ago
3	Serious illness and/or hospitalisation	N/A	– 3	– 2	– 1	0	+ 1	+ 2	+ 3	___________ months ago
4	Serious illness and/or hospitalisation of a loved one	N/A	– 3	– 2	– 1	0	+ 1	+ 2	+ 3	___________ months ago
5	Change to living conditions (e.g. moving to a new home)	N/A	– 3	– 2	– 1	0	+ 1	+ 2	+ 3	___________ months ago
6	Change in work conditions (e.g. working from home, using PPE)	N/A	– 3	– 2	– 1	0	+ 1	+ 2	+ 3	___________ months ago
7	Job loss or income loss (mine or partner’s)	N/A	– 3	– 2	– 1	0	+ 1	+ 2	+ 3	___________ months ago

8	Financial difficulty (mine, partner's, or household's)	N/A	– 3	– 2	– 1	0	+ 1	+ 2	+ 3	__________ months ago
9	Major change in social activities (e.g. spending time with friends, socialising)	N/A	– 3	– 2	– 1	0	+ 1	+ 2	+ 3	__________ months ago
10	Major change in leisure activities (e.g. sports, games, hobbies)	N/A	– 3	– 2	– 1	0	+ 1	+ 2	+ 3	__________ months ago
11	Major change in religious activities (e.g., church/ mosque/ temple attendance, prayer, worship)	N/A	– 3	– 2	– 1	0	+ 1	+ 2	+ 3	__________ months ago
12	Major change in sleeping habits (e.g., sleeping better or worse, sleeping more/less in duration)	N/A	– 3	– 2	– 1	0	+ 1	+ 2	+ 3	__________ months ago
13	Major change in eating habits (e.g., new diet, cooking more/less, eating out more/less)	N/A	– 3	– 2	– 1	0	+ 1	+ 2	+ 3	__________ months ago
14	Major change in exercise habits (e.g., gym, sports, walking/ running)	N/A	– 3	– 2	– 1	0	+ 1	+ 2	+ 3	__________ months ago
15	Major change in substance use habits (e.g., drinking, smoking, other drugs)	N/A	– 3	– 2	– 1	0	+ 1	+ 2	+ 3	__________ months ago

COVID-19 Exposure Survey

1	How many times have you been tested for COVID-19?	☐ Never been tested<br>☐ Tested once<br>☐ Tested two or more times
2	If you've been tested, what were the results?	☐ All tests negative<br>☐ One test positive<br>☐ Two or more tests positive
3	If you received a positive test result, what was the probable source of your COVID-19 exposure?	☐ From a patient I was treating<br>☐ Acquired overseas<br>☐ Family/household member<br>☐ Community transmission<br>☐ Unclear / Don't know
4	How many days of work have you missed due to either getting tested or having a positive result?	___________ days missed

5	To what extent do you agree or disagree with the following statements regarding patient care?					
		Strongly disagree	Disagree	Neither agree nor disagree	Agree	Strongly agree
a	I am aware of the plan in my ward/ department/ unit to isolate patients with suspected COVID-19.	1	2	3	4	5
b	I am concerned about spreading COVID-19 to my family members.	1	2	3	4	5
c	I am confident to care for a patient with COVID-19.	1	2	3	4	5
d	I am concerned that an outbreak of COVID-19 will occur in my ward/ department/ unit.	1	2	3	4	5

6	Have you cared for a patient with COVID-19?	☐ Yes<br>☐ No<br>☐ Unsure

7	How has your workload been affected in response to COVID-19?	☐ Increased<br>☐ Remained the same<br>☐ Decreased
8	During COVID-19, has the thought of leaving your job crossed your mind?	☐ Never<br>☐ Almost never<br>☐ Sometimes<br>☐ Almost every day<br>☐ Every day

Thrive Program Attendance and Satisfaction Survey

Impact on self

	Not helpful	A little helpful	Somewhat helpful	Quite helpful	Really helpful
1. Overall, how helpful do you feel the Thrive program was in increasing your mental wellbeing (e.g., mental health, positivity, emotional health)?	1	2	3	4	5
2. Overall, how helpful do you feel the Thrive program was in increasing your physical wellbeing (e.g., vigor, fitness, sleep)?	1	2	3	4	5

3. Following the Thrive program, how much have you changed your habits in the following life areas:

	**Not at all**	**A little**	**Somewhat**	**Quite a lot**	**Very much**
**Sleep**	1	2	3	4	5
**Exercise**	1	2	3	4	5
**Nutrition**	1	2	3	4	5
**Stress management**	1	2	3	4	5
**Social connection**	1	2	3	4	5
**Intellectual challenge**	1	2	3	4	5
**Life values/purpose**	1	2	3	4	5

3b. Following the Thrive program, how much do you intend to change your habits in the following life areas:

	**Not at all**	**A little**	**Somewhat**	**Quite a lot**	**Very much**
**Sleep**	1	2	3	4	5
**Exercise**	1	2	3	4	5
**Nutrition**	1	2	3	4	5
**Stress management**	1	2	3	4	5
**Social connection**	1	2	3	4	5
**Intellectual challenge**	1	2	3	4	5
**Life values/purpose**	1	2	3	4	5

Impact on team

	Not at all	A little	Somewhat	Quite a lot	Very much
4. Do you feel your team experienced a greater sense of cohesion and engagement with each other following the program?	1	2	3	4	5

Impact on the quality of care

	Not helpful	A little helpful	Somewhat helpful	Quite helpful	Really helpful
5. How helpful do you feel the program was in increasing the quality of care that you provide to your patients?	1	2	3	4	5

	Not at all	A little	Somewhat	Quite a lot	Very much
6a. Has your quality of care changed following the program?	1	2	3	4	5

6b. If your quality of care has changed, how? ____

Attendance and General Satisfaction with Thrive program

7. Which version of the Thrive program did you participate in?

☐ online version
☐ take-home version

8. Of the seven weeks of the Thrive program, which ones did you watch/attend or read/complete?

Week	Online Version		Take-home Version	
**1**	☐ partially watched/attended	☐ fully watched/attended	☐ partially read/completed	☐ fully read/completed
**2**	☐ partially watched/attended	☐ fully watched/attended	☐ partially read/completed	☐ fully read/completed
**3**	☐ partially watched/attended	☐ fully watched/attended	☐ partially read/completed	☐ fully read/completed
**4**	☐ partially watched/attended	☐ fully watched/attended	☐ partially read/completed	☐ fully read/completed
**5**	☐ partially watched/attended	☐ fully watched/attended	☐ partially read/completed	☐ fully read/completed
**6**	☐ partially watched/attended	☐ fully watched/attended	☐ partially read/completed	☐ fully read/completed
**7**	☐ partially watched/attended	☐ fully watched/attended	☐ partially read/completed	☐ fully read/completed

9. What did you like about the Thrive program?

____

10. What did you dislike about the Thrive program?

____

11. What other information would you like to receive on wellbeing as a staff member?

____
